# Barcoding Microtubules: Encoding Information onto Macromolecules by Photobleaching

**DOI:** 10.1021/acs.nanolett.5c00105

**Published:** 2025-03-21

**Authors:** R. Catalano, Y. Zhao, M. Pecak, T. Korten, S. Diez

**Affiliations:** † B CUBE - Center for Molecular Bioengineering, TUD Dresden University of Technology, 01307 Dresden, Germany; ‡ Helmholtz AI Team Matter, FWCC, Helmholtz Zentrum Dresden Rossendorf (HZDR), 01328 Dresden, Germany; § Max Planck Institute of Molecular Cell Biology and Genetics, 01307 Dresden, Germany; ∥ Cluster of Excellence Physics of Life, TUD Dresden University of Technology, 01062 Dresden, Germany

**Keywords:** bionanotechnology, biocomputation, barcoding, molecular motors, lab on a chip, photobleaching

## Abstract

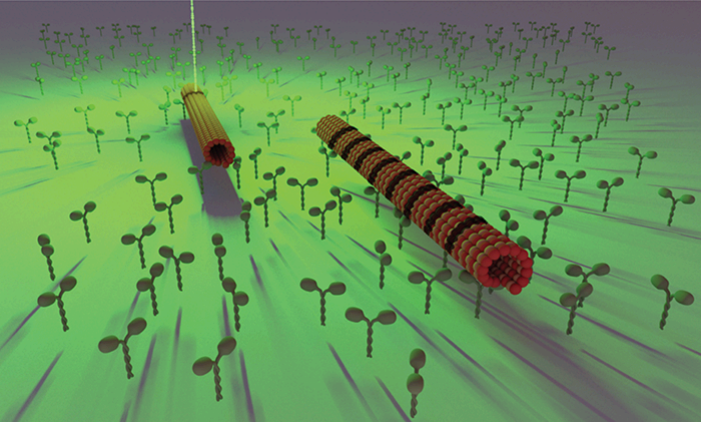

Kinesin-1-powered microtubules have emerged as versatile components in biocomputing and biosensing technologies. However, the inability to identify and track individual microtubules has constrained their applications to ensemble behaviors, limiting their potential for single-entity-based nanotechnologies. To address this challenge, we present a novel method for encoding digital information directly onto individual microtubules using photobleaching patterns. Binary numbers (1 to 15) were encoded within ∼12 μm segments of moving microtubules by photobleaching with a stationary pulsed laser, creating spatial frequency patterns corresponding to distinct bits of information. Fourier analysis enabled the accurate retrieval of the encoded data, demonstrating the feasibility of direct information storage and retrieval on macromolecular structures. This approach offers a transformative solution for recording microtubule trajectories within nanotechnological devices by encoding path information directly onto microtubules at branch points, obviating the need for video-based tracking. We anticipate that this innovation will advance the development of individualized microtubule-based technologies.

Cytoskeletal filaments and their associated molecular motors have long been explored as pivotal elements in nanotechnological applications such as biocomputation^1,2^ and biosensing.^3−6^ In particular, microtubule-kinesin systems have been successfully used for cargo loading^4,7−9^ and transport.^10−13^ The most common configuration in lab-on-a-chip devices is the gliding motility assay, where microtubules are propelled by surface-attached kinesins.^14,15^ Microtubules can be functionalized with recognition elements such as antibodies^4,16^ or DNA,^10,17^ enabling them to capture target molecules from a sample. As these functionalized microtubules glide along kinesin-coated surfaces, they transport captured molecules for detection. Precise temporal control of this system can be achieved through regulated ATP release,^18^ allowing for the coordinated movement of microtubules in biosensing applications. Several techniques have been developed to spatially guide microtubules along specific paths, including the use of surface patterning^19,20^ and nanostructures.^21,22^ While such advances have improved control over microtubule functionalization and directed gliding, current applications still primarily focus on tracking microtubule paths, without addressing the challenge of storing or retrieving information directly from the microtubules themselves. In applications such as biocomputation^7^ and sensing^21,23^ the path a microtubule takes can carry crucial information.

In 2003, Braeckmans et al.^24^ introduced a method to encode polymer microspheres using spatially selective photobleaching, creating distinct patterns identifiable via fluorescence microscopy. This technique allows for a potentially unlimited number of unique codes, significantly enhancing applications in high-throughput screening and medical diagnostics. Building upon this technique, the 2007 research by Fayazpour et al.^25^ applied these digitally encoded microparticles to pharmaceutical tablets, demonstrating their potential as a practical tool to combat drug counterfeiting by incorporating unique identifiers directly into the medication. Further advancing this concept, Huang et al.^26^ extended photobleaching-based encoding to electrospun polymer fibers, introducing another approach for embedding readable codes within pharmaceutical excipients. The broader field of photonic barcodes has been extensively reviewed by Hou et al. (2020),^27^ who summarized advancements in organic micro- and nanoscale materials for optical encoding. While initially developed for microspheres and fibers, the broader concept of encoding information via photobleaching can be extended to other systems, including cytoskeletal filaments. Given the critical role of microtubules in nanotechnological applications, exploring their potential as carriers of encoded information could open new possibilities in biocomputation and biosensing.

The state-of-the-art for recording microtubule paths involves fluorescently labeled microtubules, which are captured by time-lapse fluorescence microscopy.^28,29,21^ Their paths are then traced either manually^30,31^ or automatically^32^ from the resulting videos. Following individual filaments with these techniques is tedious and prone to errors, particularly when microtubules cross each other at shallow angles. Furthermore, this method is feasible only when the filament density is sufficiently low and when the device fits within the field-of-view of the microscope. However, more complex applications require different approaches to track individual microtubule paths. For instance, network-based biocomputation (NBC) uses microtubules that explore nanofabricated networks to solve mathematical problems.^1,33,34^ In these cases, the solution to the problem is encoded in the path that a microtubule follows to reach a target within the network. While the existence of a solution can be determined by observing whether microtubules arrive at the target exit, knowing the exact solution can require identifying the path taken by each microtubule. Reconstructing the microtubule path is feasible for small proof-of-concept networks that fit within the field-of-view of a microscope;^1,35^ however, larger networks require a method to intrinsically tag microtubules at critical junctions.^36,37^

Here, we present an approach of encoding information onto fluorescently labeled, moving microtubules by patterned photobleaching. To validate our method, we encoded all binary numbers from 1 to 15, corresponding to 4 bits of information, into about 12 μm stretches of individual microtubules. Unlike traditional approaches, our method allows for direct information encoding, enabling both storage and retrieval without the need for complete path reconstruction.

In order to encode information, microtubules fluorescently labeled with Alexa-488 were photobleached while being actively propelled by kinesin-1 motors on a glass surface. A stationary laser was used to bleach one or more lines across the moving microtubules at regular time intervals (Figure 1). The encoding procedure relies on the movement of microtubules, which translates the temporal frequency of the laser-based line-bleaching into a spatially periodic pattern of bleached spots along their length. As a microtubule moves along the *x*-axis with velocity *v*_*x*_, it traverses a focused laser beam that periodically bleaches a line along the *y*-axis. Each bleaching event occurs at time intervals *t*_*x*_ dictated by the desired spatial separation between consecutive bleaching lines *x*_b_ divided by *v*_*x*_. The laser alternates between active (on state) and inactive (off state), producing a sequence of bleached spots and unbleached regions (the “intensity profile”) on the microtubule encoding the intended information (Figure 2A).

**Figure 1:**
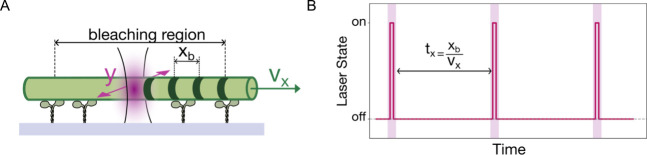
Schematics of encoding information onto microtubules. A) A microtubule, shown as a rod moving in the *x*-direction with velocity *v*_*x*_, traverses a focused laser beam that bleaches a line along the *y*-axis. The resulting bleached pattern exhibits a spatial distance *x*_b_ characterizing each spatial frequency, encoding information along the microtubule. The bleached region encompasses all the spatial frequency components. B) Bleaching events occur at time intervals determined by the microtubule’s velocity *v*_*x*_ and the desired spatial frequency *x*_b_. Each bleaching event (shaded region) lasted 5 ms, contributing to the formation of a periodic pattern along the microtubule. The laser moves in one direction along a line, bleaching it (on state), then turns off (off state) before bleaching again the next line.

**Figure 2:**
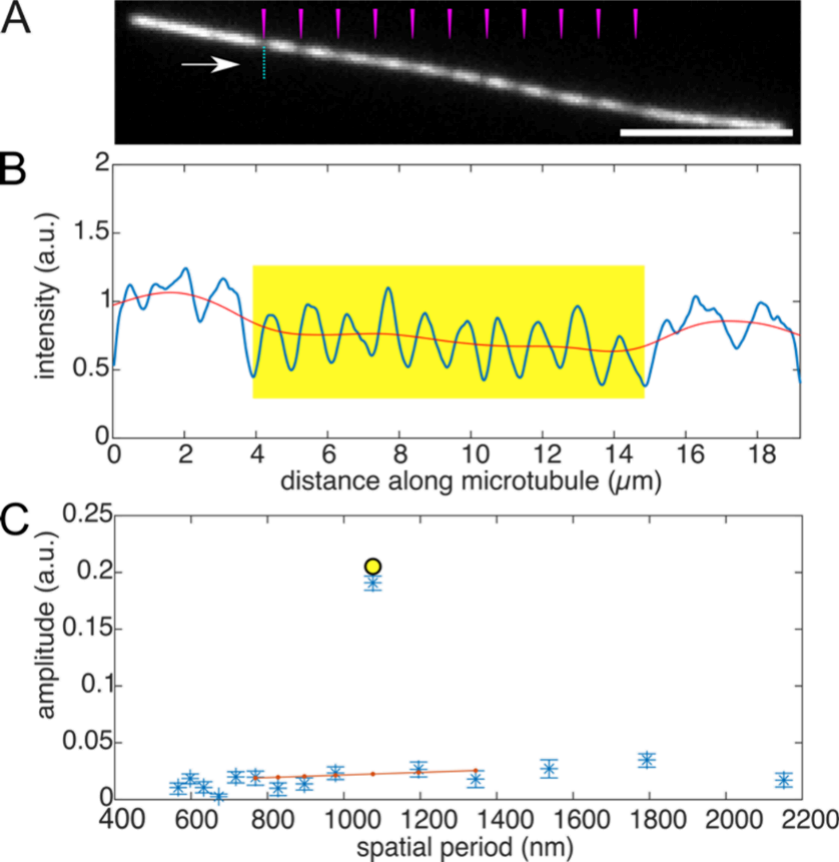
Bleaching pattern analysis. A) Fluorescence micrograph of a microtubule bleached in multiple places, indicated by purple arrowheads. Scale bar is 5 μm. The microtubule movement direction is indicated by a white arrow. B) The averaged intensity profile (bleached region is marked with a yellow box). C) Fourier transform of bleached region with a threshold line (red line) and detected spatial periodicity (yellow circle). Error bars on the detected frequencies represent the 95% confidence intervals, calculated via bootstrapping (see Supporting Information, Section 2).

The intensity profile shows a regular spatial period (Figure 2B), which was read out by Fourier analysis (Figure 2C, see Supporting Information, Section 2 for a detailed description of the analysis algorithm).

We extended the method to bleach multiple spatial periods simultaneously into the same stretch of a microtubule (see Figure 3 for examples of two, three, and four spatial periods). The resulting fluorescence intensity pattern from the different spatial periods was again analyzed by Fourier transform, enabling the identification and retrieval of multiple spatial frequencies, corresponding to the different encoded bits.

**Figure 3:**
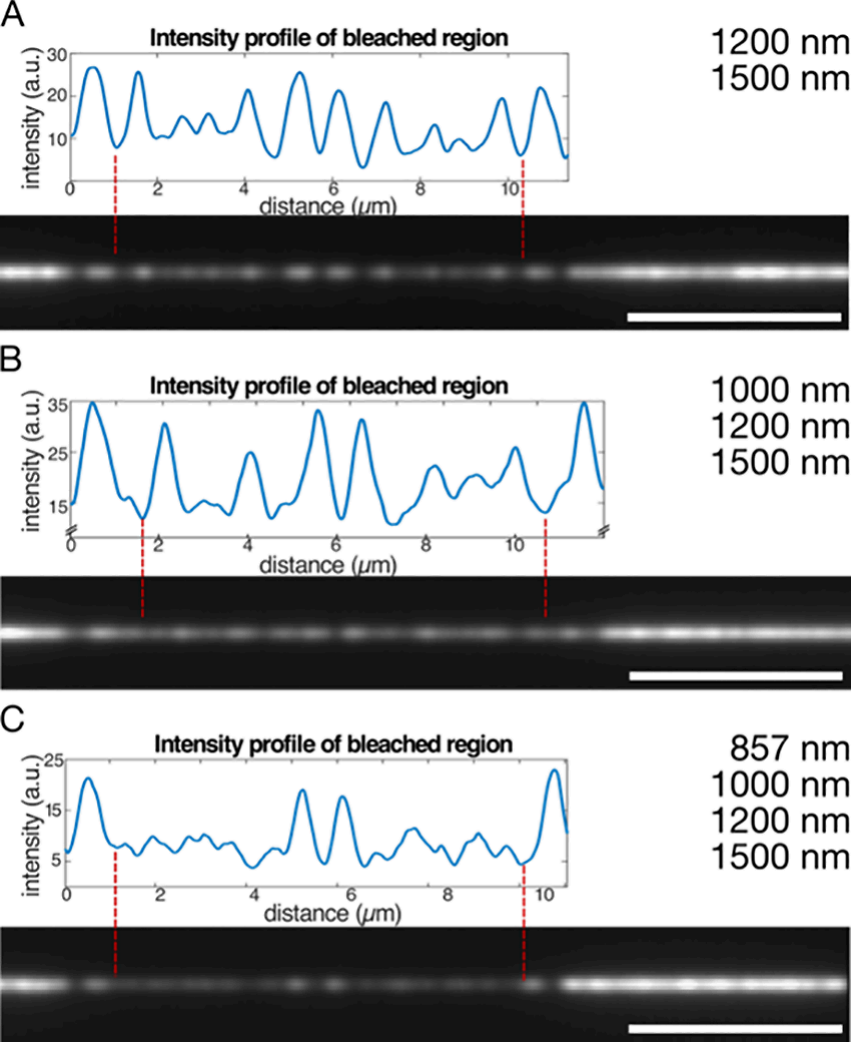
Fluorescence micrographs of microtubules bleached with overlapping spatial periods. Fluorescence micrograph of microtubules bleached with a combination of spatial periods equal to A) 1200, 1500 nm, B) 1000, 1200, 1500 nm, and C) 857, 1000, 1200, 1500 nm. The microtubules have been visually reoriented along a common axis solely for visualization purposes to provide a clearer representation of the overlapping spatial patterns. Scale bar is equal to 5 μm.

The reliability of the encoding and readout methods was tested by encoding all binary numbers from 1 to 15 using four spatial periods as four bits of information (Figure 4): spatial periods of 857, 1000, 1200, and 1500 nm were chosen based on Monte Carlo simulations (Supporting Information, Section 1). Spatial periods were assigned to bits in descending order (857 nm → 1000_2_, 1000 nm → 0100_2_, 1200 nm → 0010_2_, and 1500 nm → 0001_2_). Individual spatial periods (numbers 0001_2_, 0010_2_, 0100_2_, and 1000_2_) are clearly identifiable by eye and Fourier transform. Notably, the intensity profile for the longest spatial period is clearly not sinusoidal any more (Figure 4; upper panel for number 0001_2_), as predicted by simulations (Supporting Information, Figure S2B). This resulted in a lower amplitude of the detected spatial period (Figure 4; lower panel for the number 0001_2_). Shorter spatial periods resulted in a more sinusoidal intensity profile and in a better signal-to-noise ratio for the detected amplitudes (Figure 4; numbers 0010_2_, 0100_2_, and 1000_2_). Combinations of several bits had intensity profiles that were clearly identifiable as interference patterns, and the corresponding spatial periods were all correctly read out by Fourier transform (Figure 4; numbers, 0011_2_, 0101_2_, 0110_2_, 0111_2_, 0001_2_, 1001_2_, 1010_2_, 1011_2_, 1100_2_, 1101_2_, 1110_2_, 1111_2_). Amplitudes of spatial periods were generally lower for combinations of three or four bits (compared to one or two bits), because in these cases, the microtubules were bleached more. However, this also reduced the amplitudes of background spatial periods, resulting in a sufficient signal-to-noise ratio. Overall, the signal-to-noise ratios for all spatial periods ranged between approximately 3 (Figure 4; numbers 0001_2_ and 1111_2_) and 10 (Figure 4; number 0010_2_).

**Figure 4:**
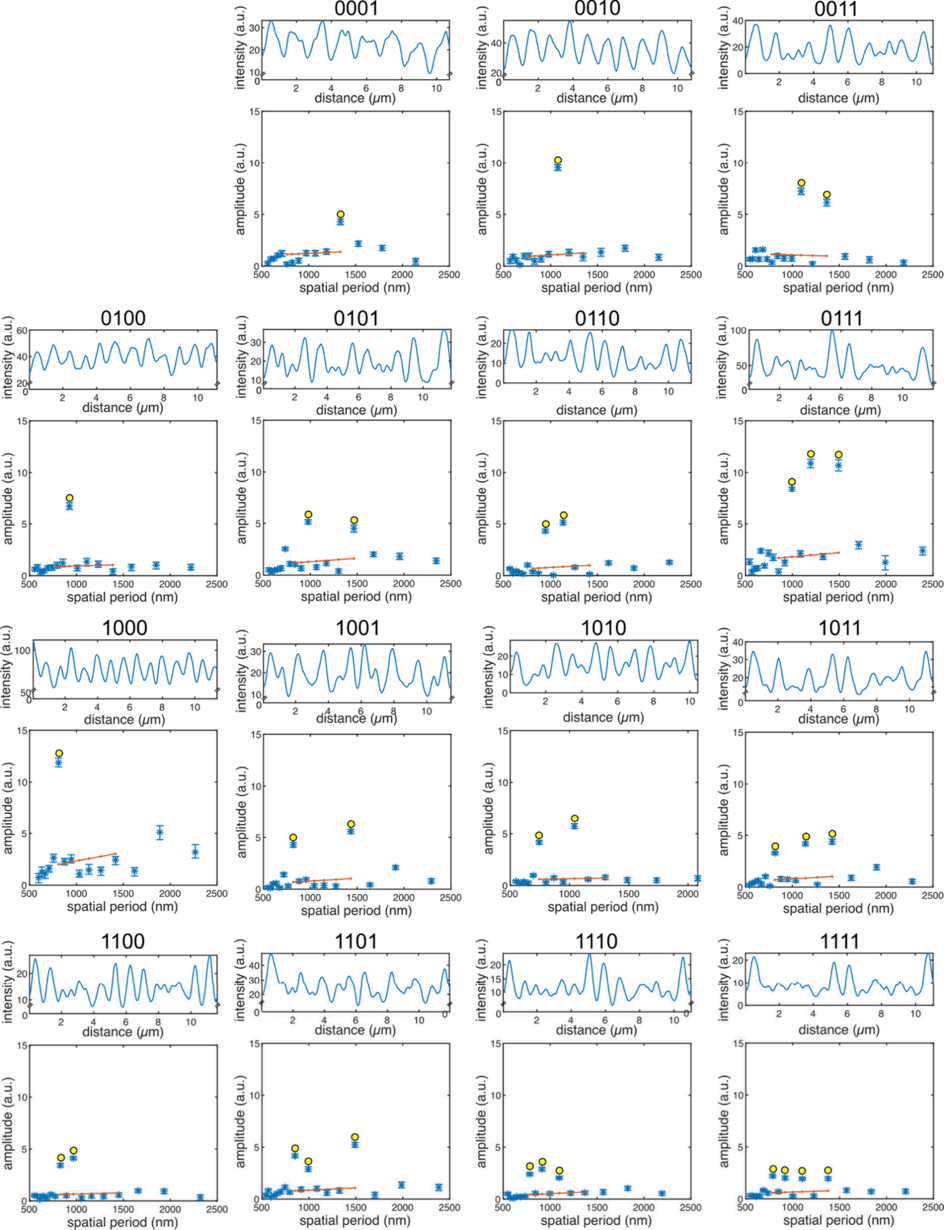
Encoding and read-out of the numbers 0001_2_–1111_2_ using up to 4 spatial periods. Microtubules were bleached with time intervals of 0.857, 1, 1.2, and 1.5 s. Due to variations in microtubule velocity, this corresponded to spatial periods in the ranges of 800–857 nm, 930–1000 nm, 1120–1200 nm, and 1400–1500 nm, which were assigned to the bits 0001_2_, 0010_2_, 0100_2_, and 1000_2_, respectively. The detected spatial periods correspond to the binary numbers given in each panel. In each panel, the upper graph shows the intensity profile of the bleached region, and the lower graph shows the corresponding detected spatial periods. The 0000_2_ case is shown in Supporting Information Figure S1. However, since the analysis algorithm requires a bleached region, we do not show it here.

In this study, we have shown how to reliably encode spatial information onto cytoskeletal filaments by photobleaching patterns on fluorescently labeled microtubules. To the best of our knowledge, this is the first record of artificially encoded digital information onto individual cytoskeletal filaments. This constitutes a significant advancement over current methods, which involve labeling populations of microtubules with different fluorophores, encoding 1 bit of information (as two different colors) but without the ability to target specific microtubules.^31,33^ A similar approach allowed for tracking individual microtubules by analyzing the speckling pattern in sparsely labeled filaments in the meiotic spindle,^38^ but did not allow targeted tagging of individual microtubules at specific locations.

For this study, we limited ourselves to spatial periods that were relatively close to the bleaching resolution. That way, we did not need to modulate the laser intensity to achieve almost sinusoidal patterns (see Supporting Information, Section 1.3 for details). Furthermore, shorter spatial periods increased the redundancy because more spatial periods fit in the bleached region. Based on our bleaching resolution of 500 nm, it should be possible to encode and decode 25 different spatial periods into the 12 μm bleached region (24, 12, ..., 0.5 μm). However, in practice, the amount of information that can be stored on a microtubule is limited by (i) the density of dye molecules, (ii) the resolution of the bleaching laser, (iii) spontaneous changes in microtubule velocity (velocity jitter), and (iv) noise. **Density of dye molecules:** The dye density limits the number of bits that can be encoded because once all dye molecules are bleached in a certain region, the microtubule cannot get darker at that point, which clips the intensity profile at lower intensities, causing information loss. Increasing the dye density would allow more information to be encoded, but the number of reactive sites on the tubulin molecule is limited by the chemistry employed. In our study, we used amino-reactive dyes which limit the total number of dye molecules per tubulin dimer to four. One alternative chemistry, thiol-reactive chemistry,^39^ is not a viable alternative, as it inhibits microtubule growth.^40^ A promising alternative is carboxy-reactive chemistry because the C-terminus of tubulin contains many glutamate residues, which can even be extended by poly glutamylation.^41^**Bleaching resolution**: The resolution of bleaching defines the shortest spatial periods that can be encoded. For bleaching, the lowest achievable spatial period is longer than the optical resolution because overlap between neighboring bleaching intensities causes a loss of fluorophores without adding information to the microtubule (see also Figure 2C). However, shorter spatial periods also increase the information density on the microtubule. Improving the resolution of bleaching could be achieved by using a higher numerical aperture objective and shorter wavelength. Additionally, a higher information density could be achieved by simultaneously labeling the microtubules with multiple fluorescent dyes that can be bleached with light of different wavelengths, allowing spectral multiplexing. **Velocity jitter**: Kinesin-1 is a stochastic motor.^42^ Therefore, the velocity of the gliding microtubules is not entirely constant. This velocity jitter causes spontaneous changes in how the temporal frequency is translated into the spatial frequency on the microtubule. This effect can be minimized by increasing the kinesin-1 motor density, because that way the random stepping of each motor largely averages out.^43^**Noise**: There are several sources of noise: (i) detector noise, (ii) shot noise, (iii) inhomogeneous labeling due to the stochastic nature of the labeling and microtubule assembly processes (speckling), and (iv) variations in the angle at which the microtubule crosses the bleaching line. We already minimized these sources of noise as much as possible with our setup: We used an EMCCD camera; thus, detector and shot noise will be hard to reduce further. We exclusively used labeled tubulin (i.e., not mixed with unlabeled tubulin) for microtubule assembly, so only an increased labeling density of the tubulin could reduce speckling further. Ideal would be if all available reactive sites on the tubulin molecules are occupied by dye molecules. Finally, we minimized angle noise by choosing microtubules that moved perpendicular to the bleaching line. Further improvements in angle noise could be achieved by guiding microtubules along the directed channels.

To encode information onto individual microtubules, other approaches based on photobleaching patterns are conceivable. One possible method is similar to one-dimensional barcodes (US patent 2612994^44^), where data are represented by varying the widths and spacings of strongly bleached regions in a digital, on–off manner along the microtubule. This would require surrounding the information-coding pattern by a start and stop region. In addition, barcoding presents particular challenges, such as sensitivity to velocity jitter and noise in the fluorescence intensity, which are less severe in our system due to inherent redundancy. However, our main motivation to use spatial periods for information encoding was to retain the possibility to extend our encoding to bleaching microtubules over their entire lengths. This would abolish the need for a common starting point for both encoding and readout. That way, it would not be necessary to detect microtubules before encoding information. Instead, the bleaching laser could continuously fire its pattern at the desired encoding location and it would be simple to add different spatial periods at subsequent bleaching stations. Thus, microtubules passing a network of channels could be encoded with information at specific locations in the network without the need to detect individual microtubules. Furthermore, many microtubules could be encoded in parallel, even if they cannot be resolved optically.

In the future, encoding spatial information about the microtubule path could be helpful to parallel processes in nanotechnological applications. In biocomputation devices, encoding information onto filaments would help downscale the network size and allow solving multiple problems on the same network.^37^ This concept applies directly to combinatorial optimization problems such as the traveling salesman problem, where the challenge is to find the shortest possible route that visits a set of cities exactly once. As the number of cities increases, the number of possible routes grows factorially, making the problem computationally intensive to solve. By encoding path information directly onto microtubules using photobleaching, a biocomputation device could simulate these paths in parallel across different microtubule routes. Each microtubule would represent a different route, and the encoded spatial information would allow the device to simultaneously explore and identify optimal routes, significantly reducing the computational time. In another example, a nondeterministic Boolean circuit network that solves a subset sum problem of combinatorial nature involving 3 numbers could be scaled down from 150 junctions^1^ to just 3 junctions^37^ applying this technique. However, we note that this improvement in the computational performance does not abolish the requirement for an exponentially increasing number of filaments. Importantly, our system adds the difficulty of reading out the encoded information from each microtubule used in the computation, which requires additional resources. These additional resources would grow linear with the number of microtubules and thus exponentially with the problem size, unless a method would be devised that can specifically detect microtubules with the correct solution among a large number of microtubules encoding nonsolutions.^37^

Similar approaches to translating a combinatorial problem into the geometry of a device can be extended to combinatorial lab-on-a-chip devices. For example, one antigen could be tested against multiple antibody clones including testing the cross reactivity of several different fluorescently labeled secondary antibodies.^45^ This would enable very fast optimization of multicolor immunofluorescence microscopy.^46^ Such a lab-on-a-chip device would feature several loading zones connected by narrow channels. The channels would guide microtubules through the loading zones such that each microtubule can randomly choose one out of several different antigens, primary antibodies, and fluorescently labeled secondary antibodies (in that order). At each loading zone, the microtubules would also be tagged with different spatial periods, so that the combination of antigens and primary and secondary antibodies that each microtubule chose can be read out. The affinity as well as cross-reactivity of the antibodies can be assessed based on the amount of secondary antibody that was picked up. That way, it would be possible to select the combination of antigens and antibodies with the highest affinity as well as the best specificity. Fifteen different tags, which is the state of the art for this study, would allow to test 2 proteins with 6 primary and 7 secondary antibodies, which would result in 82 different combinations. The advantage grows exponentially as more individual components are to be analyzed. This principle of a combinatorial lab-on-a-chip device could also be extended to other applications that require testing a large combination of components. In particular, the reconstitution of signaling^47^ or metabolic^48,49^ pathways *in vitro* could benefit from this scheme. For example, reconstituting a signaling enzyme cascade of kinases with 8 kinases and 4 different subtypes for each kinase would require testing 4^8^ = 65,536 combinations, which would require 4 × 8 = 3 tags.

In conclusion, the combinatorial nature of many biological and mathematical problems, together with the use of biomolecular agents such as microtubules that are easy to functionalize and bind to agents, paves the way for multiple applications where a tagging procedure would be relevant and desirable.

## Methods

### Preparation of Flow Channels

Microfluidic flow channels for the gliding assay were made out of Parafilm strips (2 mm wide × 8 mm long × 100 μm high). The Parafilm strips are cut and stuck between 22 mm × 22 mm and 18 mm × 18 mm coverslips, to create four flow channels. The setup was then heated to 60 °C while pressing the Parafilm to melt it and prevent any leakage. The channels were sealed before every imaging session with vacuum grease (Dow Corning).

### Microtubule–Kinesin-1 *in Vitro* Motility Assays

Microtubule–kinesin gliding assays were performed using full-length kinesin-1 and Alexa 488-labeled microtubules. Full length kinesin-1 from *Drosophila melanogaster* was expressed in insect cells and purified as described in previous studies.^50^ Tubulin was isolated from the porcine brain as described in previous studies^51^ and subsequently labeled with Alexa488.^52^ First, the surface is blocked with 20 μL of BRB80C (0.5 mg/mL casein in BRB80) for 5 min to prevent nonspecific binding. Afterward, 20 μL of a freshly prepared kinesin solution (BRB80CAD containing 0.2 mg/mL casein, 1 mM ATP, and 10 mM DTT) is introduced and incubated for 5 min to allow kinesin binding. Once the excess solution is wicked away, a motility solution containing 0.8 mM ATP, 16 mM glucose, 0.0088 mg/mL catalase, 8 mM DTT, 0.16 mg/mL casein, 0.055 mg/mL glucose oxidase, and Taxol-stabilized microtubules is added. Taxol-stabilized microtubules are previously diluted to achieve the desired density in the field of view.

### Bleaching and Imaging Methods

Photobleaching and fluorescence imaging was performed using a Nikon Eclipse Ti2 microscope equipped with a perfect focus system (PFS) and a 1.49 NA Plan Apo 100× oil immersion objective lens. The optical setup included a galvo-driven orbital module combined with a FRAP/TIRF system (iLas2, Gataca Systems) and linked to a multilaser combiner (VS-LMS-MOT100, Visitron Systems). Imagaing data were acquired using an electron multiplying CCD camera (Andor iXon DU-897z) with a 50 ms exposure time and EM-gain of 300. In gliding assays, microtubules were bleached at 60–80% laser power (9.5–12.7 mW focused to 0.6 μm × 0.6 μm, irradiance of 26–35 mW/μm^2^) and later imaged at 1% laser power in TIRF mode. Temperature for all experiments was maintained at 28 °C using an objective heater (F-25-MC refrigerated/heating circulator; JULABO GmbH). For the bleaching process, a bleaching line was defined manually by the microscope operator, using the microscope’s laser control software (VisiView). Subsequently, long (>20 μm) microtubules that crossed the bleaching line at an approximately 90-degree angle were selected by the microscope operator. Once approximately 4 μm of the microtubule had passed the bleaching line, a custom bleaching macro was executed, which calculated the time intervals necessary to encode the desired spatial frequencies (see Figure 1) and triggered the bleaching laser accordingly. The spatial frequencies to be encoded and the microtubule velocity as well as the length of the bleached region were defined as parameters in the code of the bleaching macro. The bleaching macro is available on GitHub: https://github.com/thawn/bleach-encoding.

### Image Analysis

To analyze photobleaching patterns on microtubules (see Supporting Information, Section 2 for details), we used a custom algorithm written in Matlab. First, the coordinates of individual microtubule filaments were extracted from fluorescence micrographs after the microtubules were tracked with FIESTA.^8^ These coordinates were interpolated to a 3-fold smaller pixel size, to achieve a higher resolution along the filament axis. For each frame, the intensity profile was extracted by averaging 3 pixel values perpendicular to the filament axis. These intensity profiles from multiple frames were aligned using cross-correlation, correcting for small lateral shifts that occurred between frames. This alignment process allowed us to obtain an averaged intensity profile with an improved signal-to-noise ratio.

Fluorescence micrographs were processed in two ways: (i) straightened, super-resolved images were generated by interpolating and straightening the microtubules along the *y*-axis, purely for visualization purposes to clearly represent the encoded patterns (see Figure 3), and (ii) the nonstraightened, aligned intensity profiles served as the main focus of the analysis. By aligning and averaging the intensity profiles from several frames, we ensured that the encoded patterns could be accurately retrieved and analyzed. The analysis then consisted of two main steps: identification of the bleached region and Fourier analysis. The bleached region was identified using a semiautomated algorithm that (i) fit a smoothing spline function to the intensity profile (Supporting Information, Figure S3D), (ii) identified the minimum in the derivative as the left border of the bleached region and the maximum as the right border, and (iii) manually verified that the bleached region started and ended at the first and last bleached minimum, respectively.

In the Fourier analysis, a subset of spatial periods, ranging from 850 to 1500 nm, was identified as a region of interest. First, a dynamic baseline was fit based on periods outside the subset. The program then checked whether the frequencies in the region of interest exceeded this baseline threshold, indicating significance. Bootstrapping was employed to estimate confidence intervals with a frequency considered significant only if the lower bound of its confidence interval surpassed the threshold. The analysis algorithm is available on GitHub: https://github.com/thawn/bleach-encoding.

## Supporting Information Available

Monte Carlo simulations of photobleaching microtubules; analysis algorithm (PDF)
