# Supplementary material for: Barcoding Microtubules: Encoding Information onto Macromolecules by Photobleaching
Source: Nano Lett. 2025 Mar 21;25(13):5283–90. doi: 10.1021/acs.nanolett.5c00105 (PMC11969655; doi:10.1021/acs.nanolett.5c00105)
Supplement: Supplementary file 1 — nl5c00105_si_001.pdf [file nl5c00105_si_001.pdf]

Supporting Information

Barcoding Microtubules: Encoding Information Onto Macromolecules by Photobleaching

R. Catalano[1], Y. Zhao[1], M. Pecak[1], T. Korten[2,*], S. Diez[1,3,4,*]

1. B CUBE - Center for Molecular Bioengineering, TUD Dresden University of Technology, 01307 Dresden, Germany
2. Helmholtz AI Team Matter, FWCC, Helmholtz Zentrum Dresden Rossendorf (HZDR), 01328 Dresden, Germany
3. Max Planck Institute of Molecular Cell Biology and Genetics, 01307 Dresden, Germany
4. Cluster of Excellence Physics of Life, TUD Dresden University of Technology, 01062 Dresden, Germany

* correspondence to: t.korten@hzdr.de, stefan.diez@tu-dresden.de

1 Monte-Carlo simulations of photobleaching microtubules

1.1 Simulation algorithm

For the simulations of encoded spatial patterns on microtubules, we used a custom Matlab code, which was built to model the bleaching of fluorescently labeled microtubules. The class allows for the generation of photobleaching patterns based on user-defined spatial frequencies (wave counts or wavelengths) and simulates how these patterns affect the intensity profile of the microtubule. The simulation assumes a microtubule with 13 protofilaments and a tubulin dimer length of 8 nm. Each tubulin dimer can be labeled with a maximum of 4 fluorescent dye molecules, assuming an amino-reactive dye that can either react with the n-terminus or lysin residues (see reference 52 from main text). This leads to a maximum of 52 dye molecules per 8 nm. The average number of dye molecules per nm was defined based on a labelling ratio parameter, which was 0.25 by default, resulting in each labeled dimer being assigned an average of one dye molecule, or 16.25 dye molecules per 10 nm. The total length of the microtubule was divided into smaller segments based on a simulation resolution parameter (default: 10 nm). Before each simulation, a function randomly assigned dye molecules to each segment such that the average number corresponded to the target value, but individual segments had varying numbers of dye molecules. The resulting fluorescence intensity was calculated for analysis during photobleaching experiments assuming that each dye molecule emits 100 photons on average. Based on the number of photons emitted per segment, shot noise was added to the intensity profile. Thus, the resulting intensity was calculated as follows:

$$I_m = dp + n \quad (1)$$

where I_m is the fluorescence intensity (in photons) of the respective microtubule segment, d is the randomly assigned number of dye molecules per microtubule segment, p is the average number of photons per dye molecule and n is the shot noise, which was approximated as a normal distribution with a standard deviation of $\sqrt{dp}$.

The microtubule was represented as a vector of intensities, each calculated from Equation (1). To calculate the final intensity profile, the intensity vector was convolved with a Gaussian profile with a width of the imaging resolution (default: 400 nm).

Bleaching was performed by estimating the intensity profile of the bleaching laser as a Gaussian profile with the width of the bleaching resolution (default 500 nm; see also Fig. S1). Based on the bleaching intensity, the bleaching probability for each dye molecule was calculated as follows:

$$p_b = I_b * e^{(-\frac{t}{\tau})} \quad (2)$$

where p_b is the bleaching probability for each dye molecule, I_b is the normalized intensity of the bleaching laser for the respective microtubule segment, t is the dwell time of the laser in seconds and $\tau$is the lifetime of the dye molecule (default: 0.1 s). The custom Matlab script is available on GitHub https://github.com/thawn/bleach-encoding

1.2 Measurement of the bleaching resolution

The bleaching resolution of the optical setup was experimentally measured by bleaching a single line across a moving microtubule. An acquired intensity profile (Fig. **S1**) was used to determine the bleaching resolution, defined as the full width at half maximum (FWHM), which was measured to be 500 ± 20 nm (FWHM ± SEM). This experimentally measured resolution was then used as a reference for subsequent simulations and experiments.

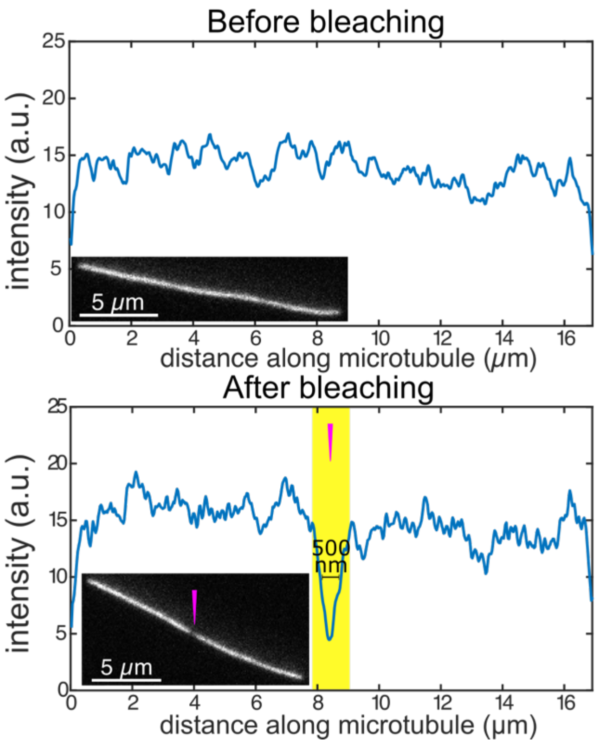

Fig. S1: Experimental determination of bleaching resolution. Intensity profiles of a microtubule before (left panel) and after (right panel) bleaching a single spot on the microtubule. Insets: Corresponding fluorescence micrographs of the microtubule. The full-width at half-maximum of the bleached line was measured to be 500 ± 20 nm (FWHM ± SEM; n=6).

1.3 Simulation results

To identify the optimal working range of spatial periods, a series of Monte Carlo simulations were performed (Fig. **S2**). Fig. **S2A-C** show simulation results for three different spatial periods: 2000 nm, 1000 nm, and 600 nm. Shown are the bleaching intensity of the laser as projected onto a moving microtubule (left) and the resulting fluorescence pattern on the microtubule after photobleaching (middle). To analyze the fluorescence patterns, we applied Fourier analysis, a technique that allowed us to decompose the observed patterns into their constituent spatial frequencies (right). The lower and upper limit for the spatial periods to be included in our bleaching range were selected based on two observations: (i) the emergence of harmonic signals in the Fourier spectrum for longer spatial periods and (ii) the overlap of bleaching profiles for shorter spatial periods.

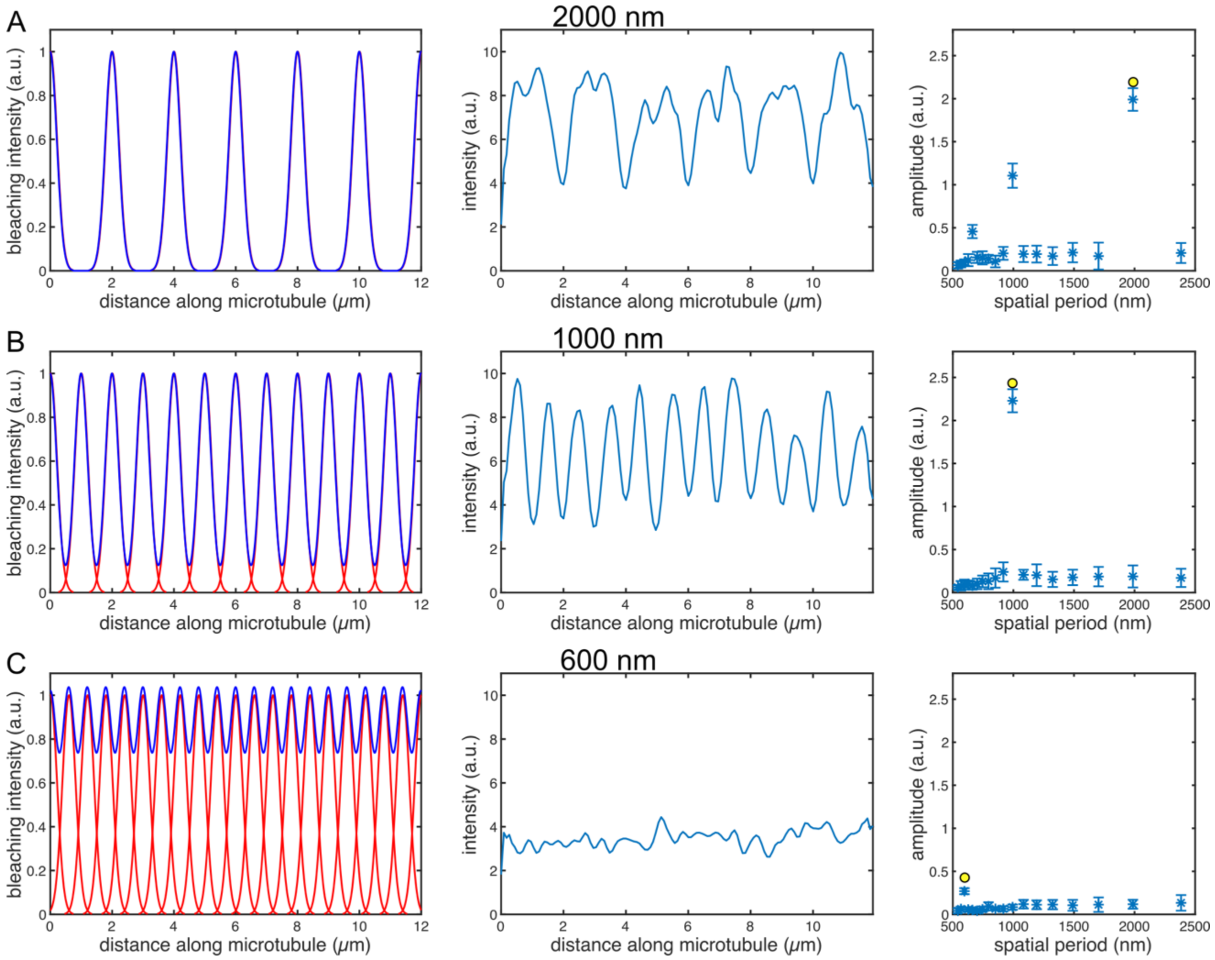

Fig. S2: Simulation of bleaching with different spatial periods. A-C) Simulated bleaching of a 12 μm long stretch of microtubule with spatial periods of 2000 nm (A), 1000 nm (B), and 600 nm (C). In brief, the simulation algorithm worked as follows: we assumed the microtubule to be a line, along which fluorophores are stochastically distributed. Each of these fluorophores had a certain probability of bleaching depending on the laser intensity at the position of that fluorophore. The laser intensity was determined by convolving the bleached positions with a Gaussian profile with the width of the experimentally measured bleaching profile. The total intensity (A-C left blue line) was the sum of the individual bleaching spots (A-C left red lines). The fluorescence intensity profile after bleaching (A-C middle) was estimated by convolving the positions of the (remaining) fluorophores with a Gaussian profile corresponding to the imaging resolution of our setup (400 nm). Spatial periods were read out by Fourier analysis (A-C right). Blue asterisks represent the mean and error bars the standard deviation of the Fourier analysis of 10 simulations. Yellow circles denote the encoded spatial period.

First, in the simulation with a spatial period of 2000 nm, the laser intensity profile showed regions with zero intensity between the bleaching lines (Fig. **S2A**, left). This occurred because the width of the bleached lines was significantly smaller than the spatial period. This created a fluorescence pattern on the microtubule that resembles a square pulse (Fig. **S2A**, middle). Consequently, Fourier analysis detected additional spatial periods corresponding to half and one-third of the original bleached spatial period, reflecting the higher harmonics introduced by the box-like shape (Fig. **S2A**, right). This effect limits the use of longer spatial periods, as the harmonic signals would overlap with other spatial periods to be potentially applied for additional information encoding. In contrast, for intermediate spatial periods (1000 nm) the laser bleaching intensities followed nicely one after the other (Fig. **S2B** left), resulting in a smooth sinusoidal fluorescence pattern on the microtubule (Fig. **S2B** middle) and a single signal with high amplitude in the Fourier spectrum (Fig. **S2B** right). However, for small spatial periods (600 nm) close to the bleaching resolution, there was a significant overlap between individual bleaching lines (red lines in Fig. **S2C** left), reducing the amplitude of the resulting bleaching profile and raising the minimum bleaching intensity (blue line in Fig. **S2C** left). Fluorophores were then bleached even in the minima of the bleaching profile (Fig. **S2C** middle) and the amplitude of the resulting spectral component was decreased (Fig. **S2C** right).

Based on these simulation results, we decided to create 8, 10, 12, or 15 bleaching events within a 12 µm region of the microtubules gliding at 930 nm/s to 1000 nm/s (at 28˚ C). Assuming the upper bound of the experimentally measured velocities, the corresponding spatial patterns then have periodicities of 1500 nm, 1200 nm, 1000 nm, and 857 nm, respectively. To achieve these spatial periods, a custom-written software controlled the time intervals between the bleaching events. The laser was programmed to turn on and off at intervals of 0.857 s, 1 s, 1.2 s, and 1.5 s. This controlled timing of the laser on/off cycle ensured the resulting spatial frequencies matched the selected periods. The resulting pattern in the fluorescence profile was analyzed by Fourier analysis, recovering the spatial periods stored in the microtubule. We note, that the above mentioned variations in the gliding velocity lead to slight variations of the inscribed spatial periods to be expected between 800 – 857 nm, 930-1000 nm, 1120 – 1200 nm and 1400 – 1500 nm. In the following, these spatial periods correspond to four distinct bits of information encoded into the microtubules.

2 Analysis algorithm

To reliably retrieve the encoded information, the spatial pattern of fluorescence intensity was read out by fitting a line to the microtubule with nanometer precision using the tracking software FIESTA[8] and plotting the intensity profile along that line (Fig. **S3A** and **B**; example of analysis for a microtubule bleached with 1200 nm spatial pattern). Due to microtubule movement, images were acquired with a short exposure time (50 ms) to reduce motion blur. To lower the impact of noise in the individual images, a stream of 40 images was acquired and the microtubule was tracked in each image (Fig. **S3C**). The resulting intensity profiles were aligned with sub-pixel resolution using cross-correlation and then averaged, resulting in a smoothed profile with increased signal to noise ratio (Fig. **S3D**). From the smoothened line profile, the bleached region from the first to the last bleached minimum was extracted (Fig. **S3D**, yellow region). The spatial frequencies were then recovered from the bleached region by the Fast Fourier Transformation (fft) function in MATLAB (Fig. **S3E**). To identify significant signals, 95% confidence intervals were estimated for all spatial periods by bootstrapping the raw intensity profiles. Each Fourier spectrum analysis was repeated 1000 times for a randomly selected set of intensity profiles (with replacement). A linear fit was then performed to the upper confidence intervals of the spatial periods that were smaller than our smallest spatial

period of interest (< 857 nm) and larger than the largest period of interest (>1500 nm). A section of this linear fit in the range of our spatial periods of interest is plotted as a red line in Fig. **S3E**. Any spatial period for which the lower confidence bound of the amplitude was above that line was deemed significant. This demonstrates that single frequencies can be reliably distinguished from background noise. The bootstrapping process ensures that the detected frequencies are statistically significant, reducing the likelihood of false positives.

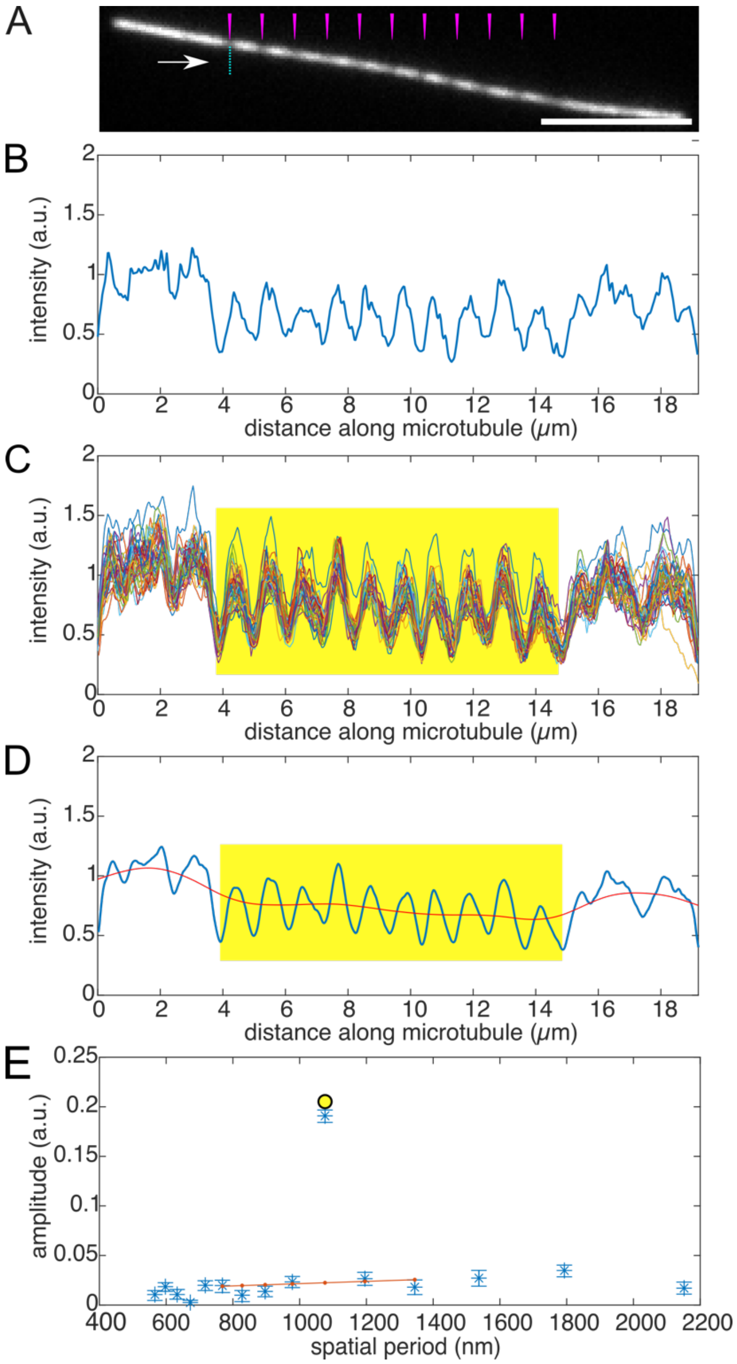

Fig. S3: Analysis algorithm. A) Fluorescence micrograph of a microtubule bleached in multiple places, indicated by purple arrow heads. The microtubule movement direction is indicated by a white arrow. Scalebar 5 µm. B) Intensity profile along the microtubule. C) Aligned intensity profiles from 40 frames. D) The averaged intensity profile (bleached region is marked with a yellow box). E) Fourier transformation of bleached region with a threshold line (red line) and detected spatial periodicity (yellow circle). Error bars on the detected frequencies represent the 95% confidence intervals, calculated via bootstrapping.
